# A Suspected Pelvic Aneurysmal Bone Cyst in Pregnancy

**DOI:** 10.1155/2013/676087

**Published:** 2013-04-16

**Authors:** Rayan Elkattah, Brooke Foulk

**Affiliations:** Department of Obstetrics and Gynecology, Quillen College of Medicine, East Tennessee State University, 325 N State of Franklin Rd, Johnson City, TN 37604, USA

## Abstract

Albeit rare, the majority of identified bone lesions in pregnancy spare the pelvis. Once encountered with a pelvic bone lesion in pregnancy, the obstetrician may face a challenging situation as it is difficult to determine and predict the effects that labor and parturition impart on the pelvic bones. Bone changes and pelvic bone fractures have been well documented during childbirth. The data regarding clinical outcomes and management of pregnancies complicated by pelvic ABCs is scant. Highly suspected to represent an aneurysmal bone cyst, the clinical evaluation of a pelvic lesion in the ilium of a pregnant individual is presented, and modes of delivery in such a scenario are discussed.

## 1. Case Illustration

**Figure 1:**
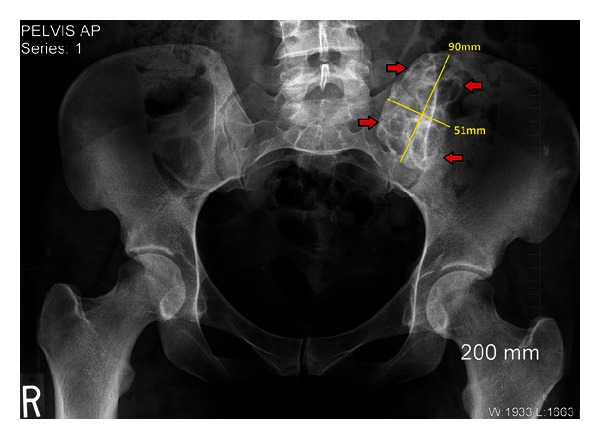
Plain radiography of the pelvis: “honeycomb” appearance—mixed lytic and sclerotic lesion (depicted within the red arrows) in the superomedial aspect of the left ilium, grossly measuring 5.1 × 9 cm.

**Figure 2:**
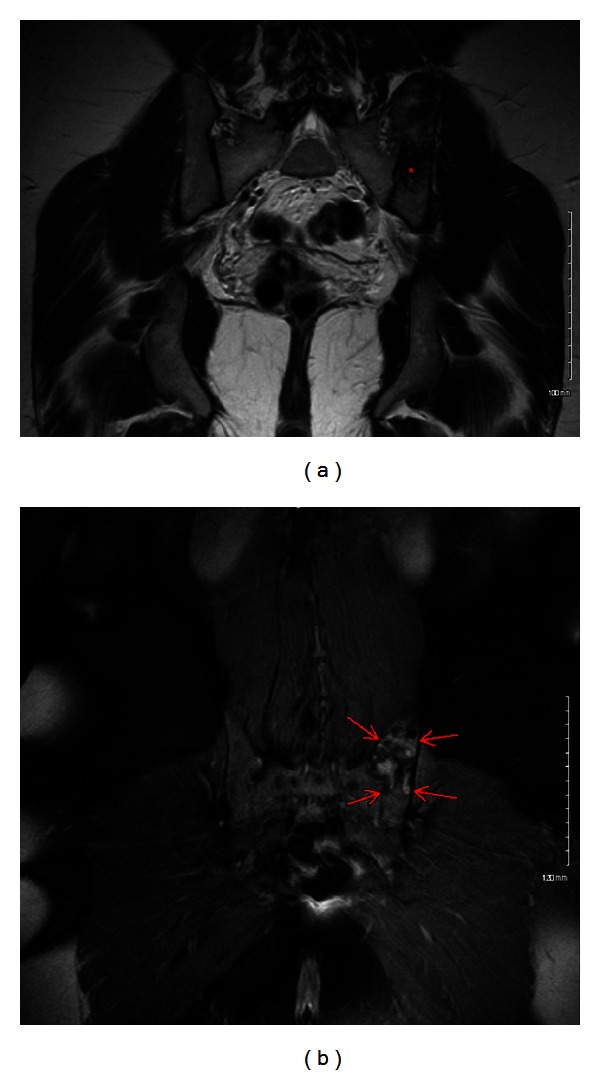
(a) Coronal T2-weighted MR image with gadolinium contrast. Lytic area within the bone lesion is noted with red asterisk. (b) Coronal T2 fat-saturated MR image without contrast. Mixed lytic/sclerotic lesion (red arrows).

**Figure 3:**
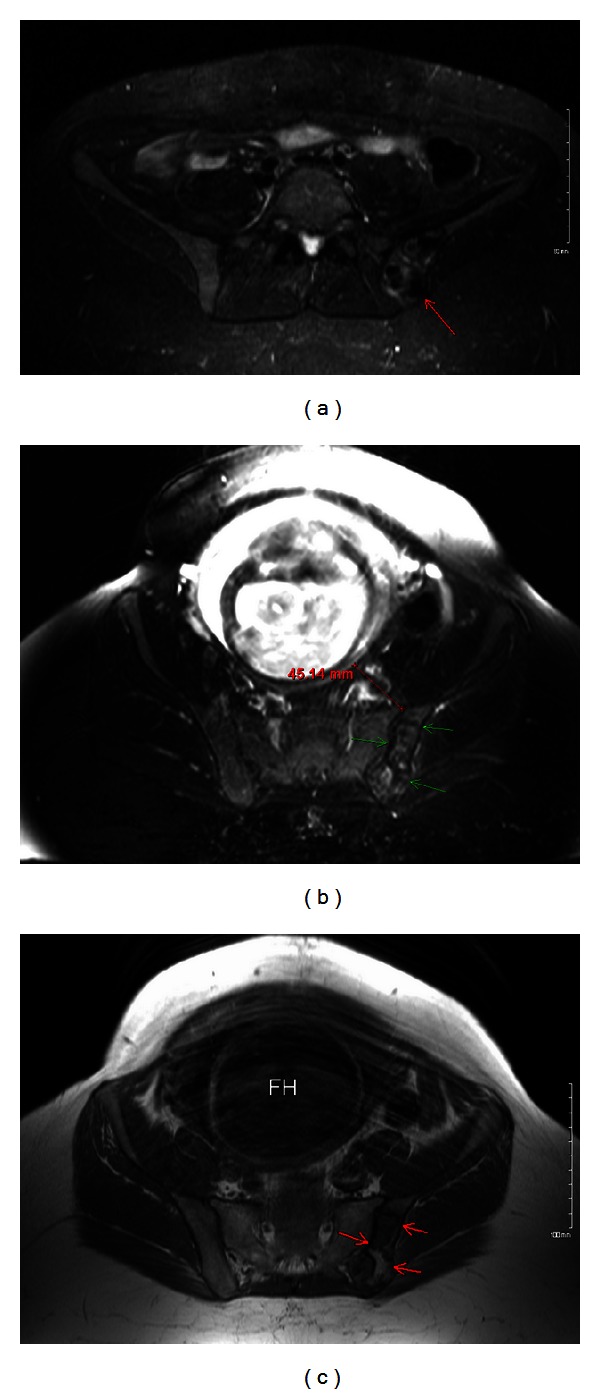
(a) Short tau inversion recovery (STIR) MR image showing lesion with gadolinium contrast. (b) T2 fat-saturated MR image without contrast. Green arrows indicate the bone lesion. The fetal head (FH) is also seen 4.5 cm away from lesion. (c) T1-weighted MR image without contrast. Red arrows indicate the bone lesion. The fetal head (FH) is also seen.

Our patient is a 26-year-old primigravid, obese, otherwise healthy female. She had originally presented to our obstetrics clinic as a transfer of care by recommendations from her primary obstetrician. The patient had been diagnosed with a left ilium bone lesion 2 years prior to pregnancy. This was discovered as an incidental finding on computed tomography (CT) and plain radiography imaging of the pelvis performed during a work-up for low abdominal and pelvic pain in an emergency department visit. On CT without contrast, this bone lesion was in the superomedial aspect of the posterior left ilium, had mixed lytic and sclerotic components, was enlarged, and abutted but spared the sacroiliac (SI) joint. Plain radiography of the pelvis (Figure 1) revealed that the lesion measured 5.1 × 9 cm, had well-defined margins, had neither cortical destruction nor any periosteal elevation, and was free of any acute fracture lines. The patient established follow-up care with an orthopedic surgeon who recommended a noncontrast magnetic resonance (MR) image of her pelvis. The MR image reaffirmed the prior imaging results, with the lesion having dimensions of 6.4 cm × 3.1 cm × 6.3 cm, abutting but not crossing the SI joint, had an intact cortex, and had mixed signals on different MR imaging modes (Figures 2 and 3). The findings were highly suggestive of an aneurysmal bone cyst (ABC). The differential diagnosis made at that time included fibrous dysplasia and benign or low-grade malignancy. After proper counseling, the patient decided to follow up the lesion annually with MR imaging since it was asymptomatic. She was advised against getting pregnant as the effects of a gestation could not be predicted, and it would be prudent to minimize the possibility of fracturing the thin cortical aspect of the ilium, a scenario that could theoretically happen with the expulsive forces of labor and thus predispose the patient to uncontrolled bleeding in her ilium: a possibly life-threatening scenario.

Her prenatal care was otherwise uncomplicated and uneventful. Her initial visit was at 9 weeks and 5 days of gestation, at which she had a dating scan performed that was consistent with her last menstrual period. Throughout all her antenatal visits, physical exam and interval history were obtained and were normal. The patient's orthopedic surgeon recommended a cesarean section as a plan for delivery with hopes that it would preclude the lesion from fracturing. In correspondence and consultation with the orthopedic surgeon, a repeat MR image of the patient's lesion was performed at 35 weeks of gestational age. On her repeated MR image, the lesion appeared to have shrunk in all of its three dimensions measuring 6.1 cm × 2.9 cm × 6.0 cm, a 15% reduction in total volume of the lesion. The patient presented at 39 weeks of gestational age for her recommended cesarean delivery. She underwent a low transverse cesarean delivery, delivering a male infant with an Apgar score of 8 and 9 at one and five minutes, respectively, and weighed 3286 grams. Intraoperative assessment of the left ilium within the pelvic cavity was attempted and was essentially normal to palpation. Hemorrhagic lesions on both ovaries were noted at the time of cesarean delivery and were biopsied. The pathology report returned as endometriotic implants. Her postoperative course was uneventful, and she was discharged home with her infant on postoperative day 2 in a stable condition with instructions to follow up in 2 and 6 weeks for an incision and postpartum check, respectively.

Her abdominal incision was healing well, and her interval history was uneventful on follow-up examination with no complaints of any pelvic bone pain. She had opted to breast feed and desired long-term birth control. A levonorgestrel intrauterine device was inserted 3 months postpartum, and the patient was released from our care with instructions to follow up with her primary obstetrician and orthopedic surgeon. 

## 2. Background

As mentioned by Mintz et al, aneurysmal bone cysts (ABCs) were first described by Jaffe and Lichtenstein in 1942 [1]. They are non-neoplastic rare bone lesions that may affect any bone [2], and represent around 1% of all benign bone tumors. They mostly occur during the second decade of life, and the ratio of female to male is 2 : 1 [2]. Seventy-five percent of ABCs are found in the long bones [3] particularly of the lower extremities [2], and the pelvis accounts for around 8–12% of all ABCs. Most occur as de novo lesions (70%) or as secondary lesions arising within other osseous conditions (30%) [4], namely, unicameral bone cysts, fibrous dysplasia, osteogenic sarcomas, nonossifying fibromas [3], osteoblastoma, and chondroblastoma [2]. ABCs may attain considerable sizes in the pelvis [5], extending into endopelvic soft tissues before becoming clinically recognizable [5, 6]. Extension beyond the thinned cortex is uncommon; however, with the growth of the lesion, there is progressive bone destruction [3]. ABCs can manifest with mass effects such as swelling, gradually increasing pain [2], neuromuscular weakness, paresthesias, and occasionally pathological fractures with lactation as a predisposing factor [7]. Obstructed labor with an oblique fetal lie necessitating cesarean delivery has also been reported as a complication of an ABC in pregnancy [5].

The true cause of ABCs is unknown. Many hypotheses have been proposed to explain the etiology and pathogenesis, and until very recently, the most commonly accepted idea was that ABCs arise as a result of hemodynamic changes within arteriovenous anastomoses with venous outflow obstruction [8] that results in dilation and rupture of the local vascular network within the bone. Further studies have uncovered a genetic component in the development of ABCs [9, 10]. On gross examination, an ABC is like a blood-filled sponge with a thin periosteal membrane. Soft, fibrous walls separate spaces filled with friable blood clot [2]. Microscopically, it appears as cystic spaces filled with blood, with fibrous septa resembling trabeculae of immature woven bone as well as hemosiderin-laden macrophages, fibroblasts, capillaries, and giant cells. 

MR imaging is the most effective modality to visualize ABCs but is rarely the first used. On plain radiography, ABCs are usually eccentrically placed and appear osteolytic. The periosteum is elevated, and the cortex is eroded to a thin margin. The expansile nature of the lesion is often reflected by a “blow-out” [11] or “honeycomb” appearance on plain radiography [3]. CT and MR imaging scan can also help delineate lesions and narrow the differential diagnosis when plain film imaging is inadequate. MR imaging can narrow the differential diagnosis of ABC by demonstrating characteristic multiple fluid-fluid levels within a multiloculated nonhomogenous lesion best seen on T2-weighted images; however, this finding is not pathognomonic for ABC [3]. The thin cortical osseous shell is represented by a low signal rim encircling the cystic lesion on T1- and T2-weighted images. 

Multiple treatment options are available when dealing with an ABC. They have the potential to spontaneously regress [2] and heal after simple biopsy [3]; however, this is rare. Selective arterial embolization can be used as a definitive treatment especially in ABCs of the pelvis, avoiding surgical exposure of deeply situated lesions and the potential for significant blood loss and death [12]. Chemical adjuvants used with grafting and curettage may be used as well [3]. En bloc resection is reserved for long bones, and irradiation is avoided secondary to the increased risks of sarcoma and the deleterious effect on gonadal tissue. Despite therapy, recurrence is seen in approximately 20% of cases.

## 3. Discussion

The data regarding clinical outcomes and management of pregnancies complicated by pelvic ABCs is scant. A comprehensive review of the English literature using PubMed yielded infrequent reports of ABCs in pregnancy. With the exception of a few case reports [5, 13], pregnancy has little to no effect on the clinical behavior of bone tumors in general. However, in our case report, the suspected ABC showed partial regression represented by a 15% volume reduction, but it is unclear whether this occurred during pregnancy since there was no MR image evidence of the ABC right before conception in this patient. It is known that ABCs are extremely uncommon [1, 14, 15] and even rarer when in the pelvis [5] in pregnancy. It follows that the management of such lesions, when encountered, is as intriguing as their incidence. Successful treatment of a massive pelvic ABC has been reported in pregnancy [5], and fatal hemorrhage secondary to curettage has been documented as well [12]. Major complications of ABCs include bleeding and pathologic fractures [2, 7]. With this risk and depending on its location, the management of pelvic ABCs is challenging particularly in pregnancy. Furthermore, pelvic bone fractures, coccygeal fractures, and pelvis dislocation during childbirth have been reported [16–18]. Brandon et al. reported a 13% risk of pubic bone fracture and a 61% bone marrow edema (nonspecific stress injury within bone) on MR imaging in primiparous women after vaginal delivery [19]. This suggests that the pelvic bones are subjected to significant shearing forces and potential trauma during childbirth. Despite no histological evidence, imaging was suggestive of an ABC, and based on the orthopedic surgeon's recommendations, a cesarean delivery was performed in order to minimize the risk of fracture and intralesional bleeding in the presumed left ilium ABC. As there are no guidelines pertaining to the obstetrical and orthopedic management of pelvic bone lesions in pregnancy, it was thought to be safer for our patient to deliver through this route. 

## 4. Conclusion

This case report illustrates the challenges that obstetricians might face with pelvic ABCs and possibly other pelvic bone cysts in pregnancy. In our opinion, several factors should be considered in planning the safest route of delivery in these scenarios, particularly the location and size of these lesions. Several reports suggest that vaginal delivery may cause significant trauma to the pelvic girdle, specifically the pubis, coccyx, sacrum, and sacroiliac joint [16–19]. Thus, it might be prudent to deliver through a cesarean route to minimize ABC-related complications in such bones. Unlike the ilium ABC described by Issa et al. [5], the lesion described in our case was much smaller and did not obstruct or impinge onto the pelvic outlet, and therefore, a vaginal delivery might have been attempted safely. As there are no obstetrical or orthopedic guidelines in the management of such lesions in pregnancy, consultation with an orthopedic surgeon and proper counseling of afflicted patients is recommended.
